# MicroRNA-338 inhibits migration and proliferation by targeting hypoxia-induced factor 1α in nasopharyngeal carcinoma

**DOI:** 10.3892/or.2022.8358

**Published:** 2022-06-27

**Authors:** Ying Shan, Xingyu Li, Bo You, Si Shi, Qicheng Zhang, Yiwen You

Oncol Rep 34: 1943-1952, 2015; DOI: 10.3892/or.2015.4195

Subsequently to the publication of the above article, an interested reader drew to the authors’ attention that, for the cell migration assay data shown in Fig. 4B, the data panels representing the ‘miR-NC inhibitor’ and ‘hypoxia’ experiments appeared to contain overlapping sections, such that they may have been derived from the same original source.

The authors have re-examined their original data, and realize that Fig. 4B was assembled incorrectly. A corrected version of Fig. 4, showing in Fig. 4B the data from one of the repeated cell migration assay experiments, is shown on the next page. The authors confirm that these data continue to support the main conclusions presented in their paper, and are grateful to the Editor of *Oncology* Reports for allowing them this opportunity to publish this Corrigendum. They also apologize to the readership for any inconvenience caused.

## Figures and Tables

**Figure 4.**
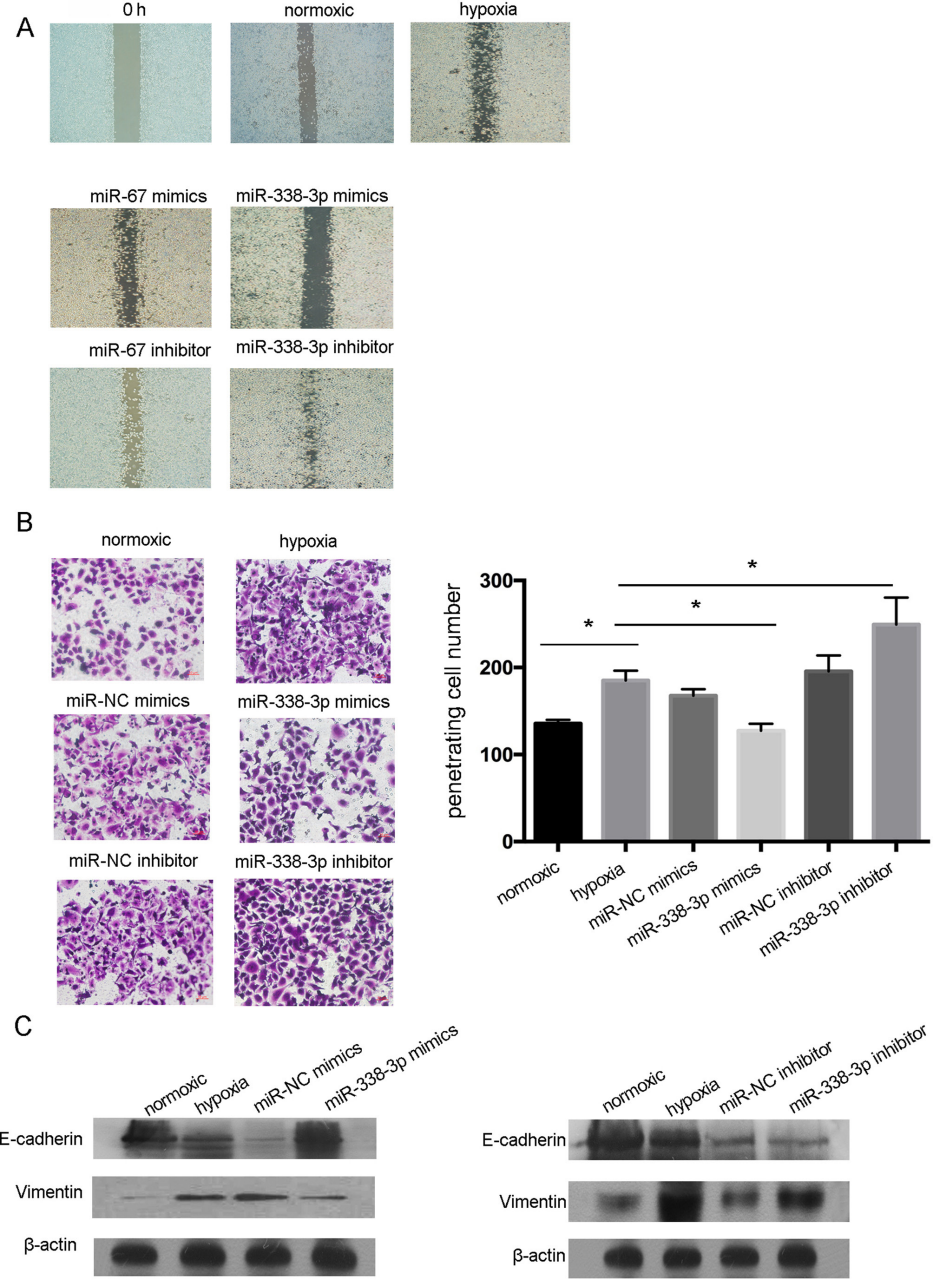
miR-338-3p regulates the metastasis potential of human NPC cell lines *in vitro*. (A) Cells transfected with miR-338-3p mimics showed a slower wound closure rate than the cells transfected with the non-specific control or miR-338-3p inhibitor under hypoxic conditions. (B) Transwell analysis confirmed the results above that the overexpression of miR-338-3p significantly reduced the metastasis capacity of the CNE2 cells under hypoxic conditions. Data are presented as mean number of migrated cells per HPF (five random fields were analyzed), compared with the control. *P<0.05. (C) Western blot analysis of E-cadherin, and vimentin expression in parental cells, miR-338-3p and miRNA control transfected cells after 24 h under hypoxic conditions compared with cells cultured under normal conditions. A representative blot from three independent experiments is shown. β-actin was used as control.

